# Real‐time dissolved carbon dioxide monitoring I: Application of a novel in situ sensor for CO_2_ monitoring and control

**DOI:** 10.1002/bit.27253

**Published:** 2020-01-11

**Authors:** Viki R. Chopda, Timothy Holzberg, Xudong Ge, Brandon Folio, Michael Tolosa, Yordan Kostov, Leah Tolosa, Govind Rao

**Affiliations:** ^1^ Department of Chemical, Biochemical and Environmental Engineering, Center for Advanced Sensor Technology University of Maryland Baltimore Maryland

**Keywords:** bioprocess monitoring and control, dissolved carbon dioxide, mini bioreactor, process analytical technology, shake flask, surface aeration intensification

## Abstract

Dissolved carbon dioxide (dCO_2_) is a well‐known critical parameter in bioprocesses due to its significant impact on cell metabolism and on product quality attributes. Processes run at small‐scale faces many challenges due to limited options for modular sensors for online monitoring and control. Traditional sensors are bulky, costly, and invasive in nature and do not fit in small‐scale systems. In this study, we present the implementation of a novel, rate‐based technique for real‐time monitoring of dCO_2_ in bioprocesses. A silicone sampling probe that allows the diffusion of CO_2_ through its wall was inserted inside a shake flask/bioreactor and then flushed with air to remove the CO_2_ that had diffused into the probe from the culture broth (sensor was calibrated using air as zero‐point calibration). The gas inside the probe was then allowed to recirculate through gas‐impermeable tubing to a CO_2_ monitor. We have shown that by measuring the initial diffusion rate of CO_2_ into the sampling probe we were able to determine the partial pressure of the dCO_2_ in the culture. This technique can be readily automated, and measurements can be made in minutes. Demonstration experiments conducted with baker's yeast and *Yarrowia lipolytica* yeast cells in both shake flasks and mini bioreactors showed that it can monitor dCO_2_ in real‐time. Using the proposed sensor, we successfully implemented a dCO_2_‐based control scheme, which resulted in significant improvement in process performance.

## Graphical abstract

A novel portable sensor for real‐time monitoring of dissolved CO_2_ (dCO_2_) in bioprocesses was developed. The measurement is based on the diffusion of dCO_2_ from the culture broth into a silicone sampling probe. The proposed sensor was successfully implemented in shake flasks and mini‐bioreactors for real‐time dCO_2_‐based control, which enables precise regulation of metabolic events in the bioprocesses and results in significant improvement in process performance.

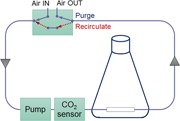

## 1 INTRODUCTION

Carbon dioxide (CO_2_) is an inevitable by‐product of respiration processes and as such always present in aerobic and anaerobic bioprocesses. This holds true for the fermentative production of various range of products using microbes or mammalian cells. CO_2_ and its hydrated counterpart ${\text{HCO}}_{3}^{-}$ are well known to interact with cellular metabolism via several ways such as (a) acidifying internal pH, (b) their role as substrate or product in various chemical reactions, (c) altering physiological properties of proteins (Blombach & Takors, 2015). In fact, it is reported that an excess of CO_2_ can act as a toxin to the culture and needs to be constantly controlled and observed (Shang et al., 2003). High dissolved carbon dioxide (dCO_2_) also leads to decreased glucose, lactate, and glutamine specific metabolite rates. Increased dCO_2_ causes perturbation in intracellular pH, thereby affecting pH‐dependent enzymatic reactions. For example, phosphofructokinase, which is a rate‐limiting enzyme in the glycolytic pathway, is inhibited by low intracellular pH. Thus, dCO_2_ has a direct impact on metabolic pathways (McIntyre & McNeil, 1997).

A dCO_2_ level above 20% is reported to be a growth inhibitory factor for certain microbial as well as mammalian cells which necessitates an effective aeration strategy during scale‐up of bioprocesses (Blombach & Takors, 2015). However, excessive stripping of dCO_2_ is also detrimental to cell growth, which suggests that there is likely an optimal level of dCO_2_ for cell culture (Mostafa & Gu, 2003). The organism used for production, the product of interest and the required quality of the product will certainly determine this optimal dCO_2_ value. The concentration of dissolved oxygen (DO) and carbon dioxide vary over time due to respiration of the cells and hence these parameters can give the true metabolic signature of the culture broth if monitored in real‐time. In addition, as an intrinsic property, partial CO_2_ pressure of shake flask, benchtop lab‐scale bioreactor, and the production bioreactor will differ significantly as it is an inherent consequence of high absolute pressure and mixing conditions. Hence, dCO_2_ concentrations must be analyzed carefully and thoroughly during scale‐up and technology transfer activities (Matsunaga, Kano, Maki, & Dobashi, 2009; Mitchell‐Logean & Murhammer, 1997; Mostafa & Gu, 2003). The real‐time dCO_2_ values will be useful not only for optimizing but also for reproducing culture conditions independent of scale (Ahuja, Shilpa Jain, & Ram, 2015). Thus, the careful monitoring and control of DO and dCO_2_ are needed in assessing the culture conditions (Ge, Kostov, & Rao, 2003; Gupta & Rao, 2003).

With Food and Drug Administration's process analytical technology (PAT) drive and rigorous quality requirements especially in biologics manufacturing (Food and Drug Administration, 2004), the attention towards continuous process supervision and real‐time control is greater than ever (Chopda, Gomes, & Rathore, 2016; Gomes, Chopda, & Rathore, 2015; Gomes, Chopda, & Rathore, 2018). Despite this fact, small‐scale processes are still lagging in terms of generating real‐time data due to a lack of suitable online sensors. Traditional electrochemical sensors are usually not used because they are bulky and invasive. Disposable optical sensors are small and only partially invasive, but there are concerns regarding the toxicity of the patch and the phototoxicity of the illuminating light (Ge & Rao, 2012; Ge, Kostov, & Rao, 2005; Gupta & Rao, 2003). Over the past decade, new sensor technologies have become available to monitor the performances of shake flasks and small‐scale fermentation systems (Table 1). Among these, the oxygen transfer rate (OTR) device and respiration activity monitoring system (RAMOS) measure OTR and respiration activities (not direct O_2_ and CO_2_ concentration) in the headspace of a shake flask and not the dissolved CO_2_. Furthermore, these systems use two pipes inserted instead of culture plug with ports for inlet and outlet for aeration and exhaust, respectively (Anderlei & Büchs, 2001; Anderlei, Zang, Papaspyrou, & Büchs, 2004; Scheidle, Klinger, & Büchs, 2007; Seletzky et al., 2007). Another system, BCpreFerm can measure O_2_ and CO_2_ concentration in the headspace of shake flask with a culture plug but not the dissolved CO_2_ (BlueSens gas sensor GmbH). In all these three systems sensors or a measuring device rotates with the shake flask which may sometime cause drifts in the signal. The fluorescence‐based method has been reported to measure O_2_ and CO_2_ in the headspace as well as in culture broth. The effect of fluorescent pigment has always been debated (Ge et al., 2005; Gupta & Rao, 2003). Takahashi et al. developed a circulation‐based direct monitoring and sampling system with a circulation bypass component in the measuring site tilted in such a way that cells do not accumulate and the air bubbles are well dispersed (Takahashi & Aoyagi, 2018a, 2018b; Takahashi, Sawada, & Aoyagi, 2017). However, it is critical to prevent cells from clumping to avoid the clogging of the liquid circulation part.

**Table 1:** Monitoring devices for shake flask cultures

Phase	Sensor name/description	References
Liquid (culture broth)	SENBIT system—uses Clarke type electrode to measure O_2_ and pH	Vasala et al. (2006)
	Fluorescent illuminator & detector—immobilized optical sensor used to monitor O_2_ and pH	Flitsch, Ladner, Lukacs, and Büchs (2016)
		Gupta and Rao (2003)
		Schneider, Schütz, John, and Heinzle (2010)
		Tolosa et al. (2002)
		Wittmann, Kim, John, and Heinzle (2003)
	Bioprocess monitoring 60—measures O_2_ and pH	Kuhner: BPM‐60
	InPro®5000, Mettler‐Toledo	Chen et al. (2008)
		Srinivasan, Feng, and Lin (2012)
	Fiber optic dCO_2_ sensor‐YSI 8500	Lander and Kruglyak (1995)
	Kurt‐Schwabe‐Institut Meinsberg electrochemical CO_2_ probe	Frahm et al. (2002)
	In situ sensor—diffusion rate‐based measurement	Chatterjee et al. (2015)
Gas (headspace)	OTR device—measures OTR in the headspace	Anderlei and Büchs (2001)
	RAMOS—measures OTR in the headspace with penetrating the optical sensor	Anderlei et al. (2004)
	BCpreFerm system for shake flasks—dual CO_2_ and O_2_ sensors that attach to two outlets on a tri‐outlet shake flask. Infrared‐based measurements	BlueSens gas sensor GmbH
Liquid and gas	Used RAMOS to measure in the headspace and rotating flexi‐tube optical sensor to monitor dissolved O_2_, CO_2_, and culture pH	Hansen, Jacob, Luchterhand, and Büchs (2012)
	Fluorescence‐based method for monitoring CO_2_ and O_2_ in the culture broth and in the headspace of an Erlenmeyer flask and broth pH as well	Ge and Rao (2012)
	Circulation direct monitoring and sampling system—a system that continuously extracts and measures liquid (by electrode) and gas‐phase samples	Takahashi et al. (2017)
		Takahashi and Aoyagi (2018a, 2018b, and 2018c)

Abbreviations: dCO_2_, dissolved carbon dioxide; OTR, oxygen transfer rate; RAMOS, respiration activity monitoring system.

In this article, we describe a novel method that correlates the concentration of dissolved CO_2_ in culture broth with the initial diffusion rate of the CO_2_ across a gas‐permeable tubing. Silicone tubing was fully immersed in the culture medium through which CO_2_ gas diffuses which was then circulated to a CO_2_ sensor equipped with a custom‐designed microcontroller pump and valves, located outside of the cell culture vessel. We have decided to select two model organisms for this study having distinct respiration activities. Baker's yeast was selected because it is well‐known for its Crabtree effect. Through its Crabtree or overflow metabolism, yeast cells can switch from purely oxidative metabolism to a respiro‐fermentative metabolism even under fully aerobic conditions as soon as glucose exceeds the critical concentration of about 0.1 g/L (Kasperski & Mis ´kiewicz, 2008). On the other hand, we have also selected a recombinant yeast *Yarrowia lipolytica P01g‐Leu*, which is Crabtree‐negative organism. The details of strain and culture conditions are given in the respective section.

The implementation of our novel sensor in monitoring dCO_2_ in shake flask and in mini bioreactors was demonstrated using yeast fermentation as a case study. We were able to demonstrate the distinct dissolved CO_2_ profile for these selected two organisms and further demonstrated a control strategy to control the metabolism. We believe that the enabling PAT at such small scale of operation will allow users to develop a scaled‐down model of pilot‐scale bioreactor operations. This will not only make the process development cost‐effective and faster but also expected to follow a smoother transition of scale‐up and technology transfer activities.

## 2 MATERIALS AND METHODS

### 2.1 Dissolved CO_2_ sensor

**Figure 1:**
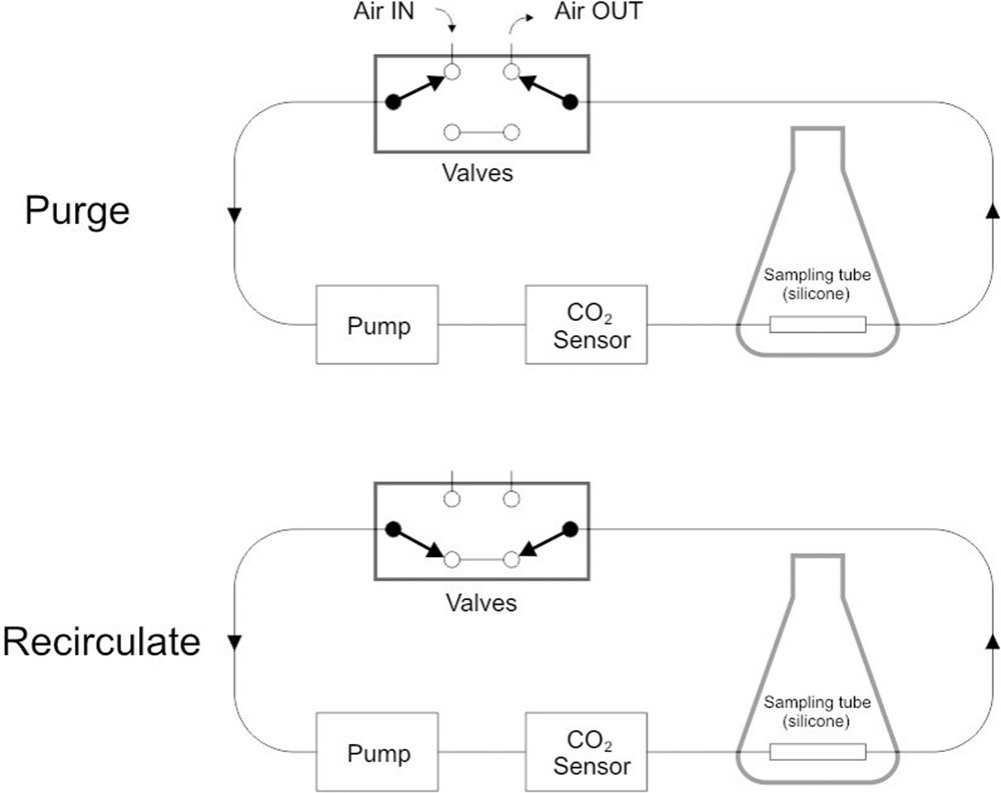
Illustration of the operational working of the dissolved carbon dioxide sensor. In the purge mode, the ambient air outside the culture vessel was pumped into the system (Air IN) and allowed to flow out through a separate outlet (Air OUT). This process purges any CO_2_ in the tubing system and returns it to normal atmospheric levels (~400 ppm). On the other hand, in recirculation mode, the Air IN valve closes, allowing CO_2_ from the culture to diffuse across the gas‐permeable tubing into the system. The pump recirculates the diffused gas through the CO_2_ sensor

**Figure 2:**
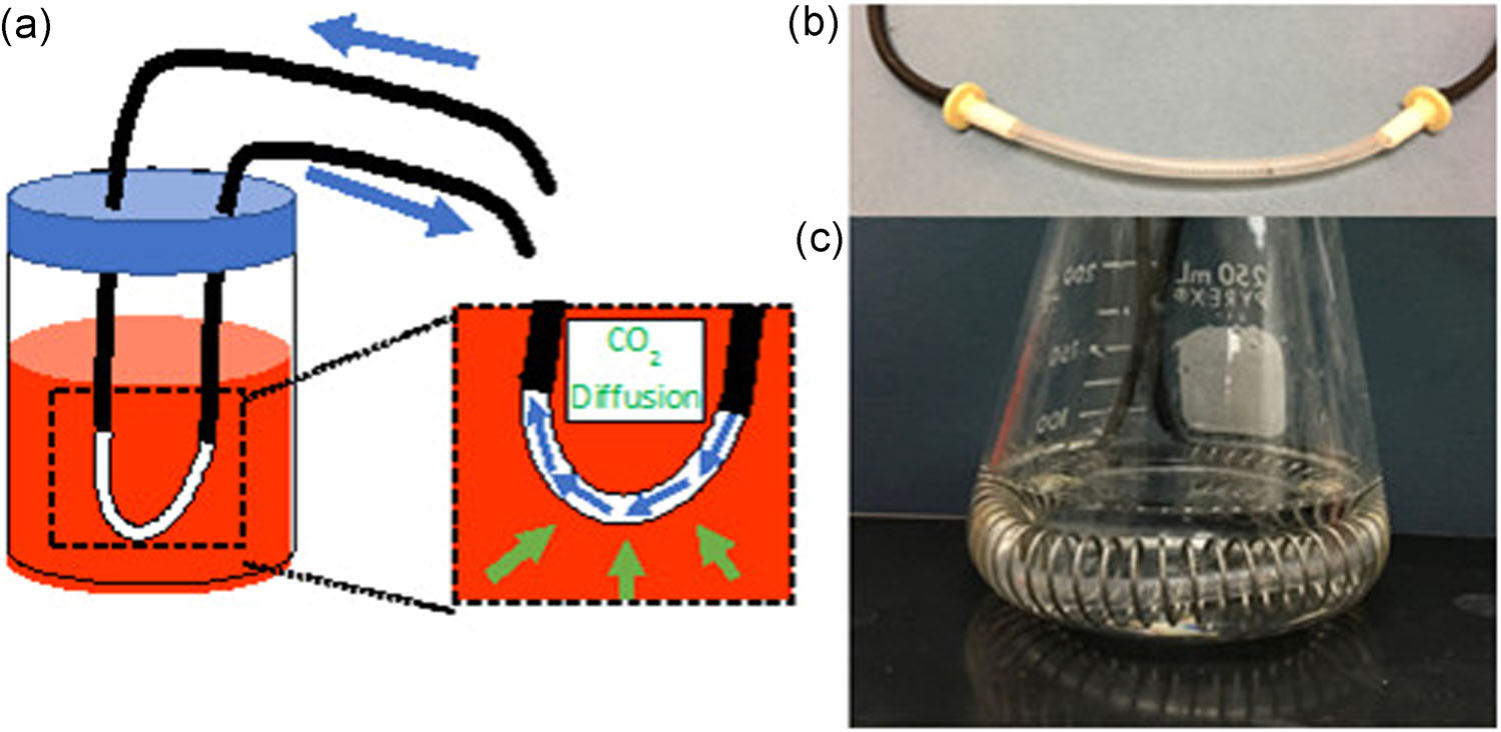
(a) Close‐up view of airflow within the silicone sampling tube for dissolved carbon dioxide measurements inside a flask. CO_2_ in the culture diffuses across the silicone sampling tube (green arrows) and enters the flow of air (blue arrows), which comes out of the sampling tube and returns back to the sensor. (b) Close‐up view of assembled silicone sampling tube with spring inside to prevent kinking. (c) Assembled silicone sampling tube ready for measurement inside a shaker flask. The coil surrounding it prevents the tube from shifting while the flask is being shaken [Color figure can be viewed at wileyonlinelibrary.com]

**Figure 3:**
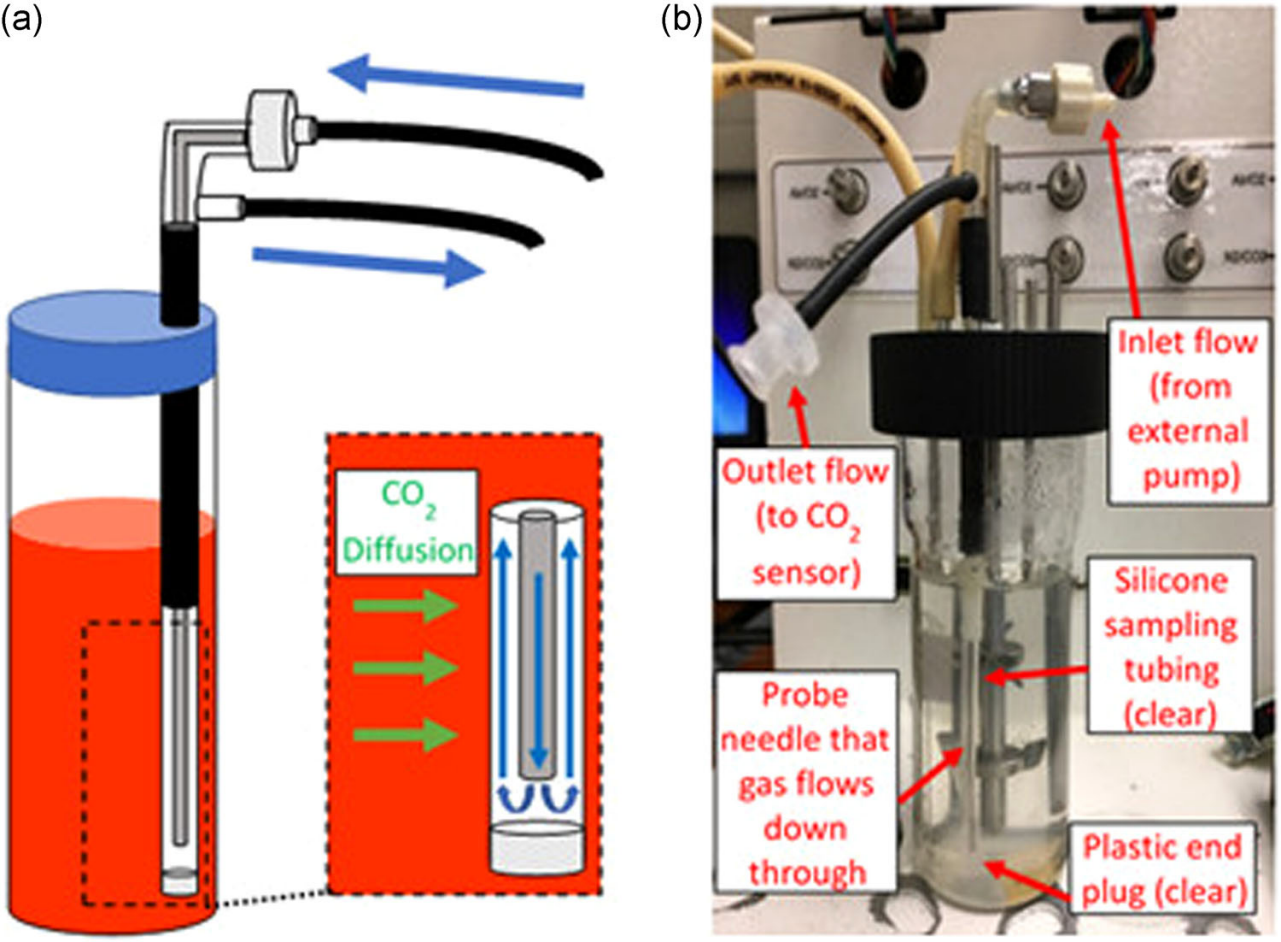
(a) Close‐up view of airflow within the probe for dissolved carbon dioxide measurements inside a mini bioreactor. CO_2_ in the culture diffuses across the silicone sampling tube (green arrows) and enters the flow of air (blue arrows), which comes out of the needle and back up into the probe housing before exiting out of the side outlet. (b) Measurement probe inside an assembled mini bioreactor vessel [Color figure can be viewed at wileyonlinelibrary.com]

This novel method correlates dCO_2_ concentration in cell culture with the initial diffusion rate of the CO_2_ across gas‐permeable silicone tubing, which was provided by Sartorius (Goettingen, Germany) and originally used for aeration in bioreactors (BB‐8848017). Silicone tubing (ranging from 4 to 6 cm in length) allows for the diffusion of CO_2_ across its wall when fully immersed in the culture medium and connected to a CO_2_ sensor (K30 CO_2_ sensor from http://CO2Meter.com). The sensor is a fast‐response infrared CO_2_ sensor, which has a measurement range of 0–10,000 ppm with a precision of 20 ppm. The sensor is designed to output digitized values in serial format (RS 232). It is connected to a computer via serial‐to‐USB converter (i.e., FT232 from Future technology). The system is equipped with two 3‐way valves and a pump, which are located outside of the cell culture vessel. The gas pump is a microdiaphragm pump produced by Parker (Hollis, NH), which provides a flow rate of up to 800 ml/min. The 3‐way valves are solenoid valves produced by the Lee Company (Westbrook, CT). They require only a very small power to operate (~3  mWh/actuation), have small dimensions (<2″ in any direction). Versilon F‐5500‐A tubing, which is reported to have significantly low CO_2_ diffusivity, was used to connect the system components together (shown as black lines in Figures 1, 2, 3). The electronics for activating the valves and digitizing the readings of the sensors are controlled by a dedicated microcontroller, which is also responsible for communicating the data to the computer (Ge et al., 2018). The program is written in LabVIEW, which controls the valves, reads the sensors, logs the data, and calculates the slopes in real‐time. All the time intervals are user adjustable so as to allow flexibility of the software.

Before a measurement cycle, ambient air outside the culture vessel was pumped into the system (Air IN) and allowed to flow out through a separate outlet (Air OUT). This process purges any CO_2_ in the tubing system and returns it to normal atmospheric levels (~400 ppm). After the purging process is complete, the valves close, allowing CO_2_ from the culture to diffuse across the gas‐permeable tubing into the system. The pump recirculates the diffused gas through the CO_2_ sensor. The purge and recirculate modes of operation and sensor setup are illustrated in Figure 1. The measurement principle and the operation procedure have been described in detail previously (Chatterjee et al., 2015).

Figure 2 shows the sensor design for use in a simple cell culture vessel (such as a shake flask), while Figure 3 shows the design for use in a mini bioreactor. The major difference between the two setups is in the direction of airflow through the silicone tubing. We have used a standard Erlenmeyer shake flask of 250 ml capacity with a sponge cap and 25 mm shaking diameter. For a shake flask, the silicone tubing was connected to the gas‐impermeable Versilon tubing with simple luer fittings. A spring was inserted through the tube to avoid blockages, bents or kinks. For shake flask, aseptic sampling was carried out through a Versilon tube inserted in it so as to have minimal disturbance in O_2_ and dCO_2_ balance, which might change significantly if sampling carried out by conventional way of opening the culture plug (Takahashi and Aoyagi, 2018c). A cylindrical mini bioreactor (2.5 cm inner diameter) of 100 ml capacity was used for controlled feeding experiments. For the mini bioreactor, a probe construct was used that is similar in design to typical PAT probes used in larger bioreactors. Figure 3 outlines the direction of airflow through the top of the probe, down through a needle that is surrounded by silicone tubing, back up through the needle housing, and out the side of the probe. This allows for diffusion rate measurements to be collected in a narrow culture vessel with a low volume.

### 2.2 Sensor characterization

After the prototype was built, the characteristics of the sensor such as sensitivity, the limit of detection (LOD), and so forth were studied. To avoid the accumulation of CO_2_ in the system, the purge stage should last long enough. After some initial experiments, the following durations were determined to be the most appropriate: 30 s for the purge stage and 60 s for the recirculation stage. During the recirculation stage, the sensors were read every 2 s and the readings were recorded in the computer. The initial diffusion rate (the slope of the rising CO_2_ concentration) was calculated by fitting the readings to a linear equation 15 s after the beginning of the recirculation and when there was a streak of five consecutive rising readings. After that, the slope was updated for every new sensor read until the end of the recirculation stage. The measurement cycle would repeat until it was stopped by pressing the stop button.

The calibration of the sensor was done by pumping in a known concentration of CO_2_ into a flask filled with cell culture media at the same temperature and agitation speed as what will be used for cell growth. The sensor was calibrated up to 20% dCO_2_ because most bioprocesses generally have dCO_2_ levels lower than that. The sensing range of the sensor can be extended to 100% dCO_2_ if a smaller size or less permeable sampling tube is used. For zero calibration point, the sensor was flushed with air and the reading of the sensor was considered as the baseline of the signal. The gas mixtures with desired CO_2_ concentrations were obtained by mixing pure CO_2_ and air through two mass flow controllers (Model #: 32907‐63; Cole‐Parmer Instrument Company, Vernon Hills, IL). After the gas mixture reached equilibrium with the media, the CO_2_ diffusion rate across the sampling tube was measured following the procedure described above. By repeating this process for several concentrations of CO_2_, a calibration curve could be constructed that is used to determine the dCO_2_ concentration in cell culture based on the measured diffusion rate. The presence of moisture can affect the sensitivity of the sensor and hence it is recommended that after autoclaving, keep the sensor in purge mode for 2–3 hr.

### 2.3 Strains and culture conditions

The developed sensor was used to monitor dCO_2_ in both baker's yeast and *Y. lipolytica P01g‐Leu* fermentations. DO was monitored by an optical sensor that is provided by Scientific Industries, Inc. The detailed description of the DO optical sensor can be found in the literature (Tolosa, Kostov, Harms, & Rao, 2002). The cultures were sampled once a day for optical density measurement.

#### 2.3.1 Baker's yeast fermentation

Fleischmann's Active Dry Yeast (*Saccharomyces cerevisiae* yeast, also known as baker's yeast) was used in these experiments and was directly added to yeast extract–peptone–dextrose (YPD) media incubated in shaker flasks and mini bioreactors. The media was prepared using individual components obtained from Sigma‐Aldrich. For sensor characterization (proof‐of‐concept) run, the concentrations were as: 10 g/L of yeast extract, 20 g/L of each peptone and dextrose. For the control experiments, the dextrose concentration was reduced to 3 g/L deliberately while other components were at the same concentrations. The lower initial dextrose concentration gave us the flexibility to test the CO_2_ control strategy by adding additional glucose. The initial yeast dry mass and the YPD media concentration were noted for each experiment involving this strain. Dry yeast (0.2 g) was added to 50 ml of YPD media in a shake flask and allowed to grow at 30°C at 250 rpm for 4 hr. This grown inoculum was further used to start the experiments with the desired initial inoculum density.

#### 2.3.2 *Y. lipolytica P01g‐Leu* fermentation

*Y. lipolytica* is classified as an oleaginous yeast species because of its ability to accumulate lipids in large quantities (Xu, Qiao, & Stephanopoulos, 2017). We used a genetically modified version of a leucine auxotroph strain (*P01g‐Leu*) that requires a leucine complementation pathway to grow (Xu, Qiao, Ahn, & Stephanopoulos, 2016). This strain was engineered for flavonoid biosynthesis (*P01g* with flavonoid pathway). The *P01g‐Leu* strain was cultured in the YPD media because of its inability to synthesize leucine (leucine is needed in the culture media to grow). Three hundred microliters of glycerol stock were added to 5 ml of YPD media in 50 ml Falcon tube and allowed to grow at 30°C at 250 rpm for 20–24 hr. This preculture was further used to inoculate the shake flask culture with the desired starting optical density.

## 3 RESULTS

### 3.1 Sensor characterization

**Figure 4:**
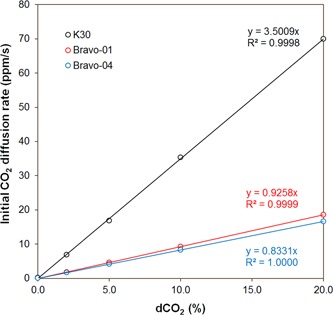
Calibration curves for three different prototypes showing the initial diffusion rate of CO_2_ through the sampling tube versus dissolved carbon dioxide concentration. K30 was built with a K30 CO_2_ sensor. Bravo‐01 and Bravo‐04 were built with a LI‐820 CO_2_ gas analyzer. The calibrations were performed in triplicate. The standard deviations are given as error bars, which are invisible because they are smaller than the symbols [Color figure can be viewed at wileyonlinelibrary.com]

Figure 4 shows the calibration curve for the K30‐based dCO_2_ sensor, which was used in the later fermentation experiments. For comparison, the calibration curves for two different dCO_2_ sensor systems built with LI‐820 CO_2_ gas analyzers (Bravo‐01 and Bravo‐04) were also provided. It can be seen that all three calibration curves fit very well to a linear equation with a negligible *y*‐intercept (correlation coefficient *R* ^2^ ≈ 1.0).

The slope of the lines represents the sensitivity of the sensor. According to our previous studies (Chatterjee et al., 2015), the sensitivity of the rate‐based sensor is affected by the mass transfer coefficient of CO_2_ in the sampling tube material (*k*), the total mass transfer area of the sampling tube (*A*), and the internal volume of the sensor system (*V*):

(1) dCdtt=0=kAVdCO2

Although the K30 CO_2_ sensor has much lower precision than the LI‐820 CO_2_ gas analyzer (20 vs. 1 ppm), the K30‐based dCO_2_ sensor has a much higher sensitivity than the LI‐820‐based dCO_2_ sensor (~3.5 vs. ~0.9 ppm/%) due to its much lower volume. Increasing the mass transfer area (the size) of the sampling tube can increase the amount of CO_2_ diffusing into the system. As a result, a higher signal will be detected at the same dCO_2_ level. Thus, the sensitivity of the system will be increased. Using a more permeable material for the sampling tube, the sensitivity of the system can be increased. However, using a too permeable material could saturate the CO_2_ sensor, resulting in erroneous readings.

The LOD of the sensor, which is the lowest dCO_2_ that the sensor can reliably measure is defined as three times of baseline signal noise divided by the sensitivity. This value was determined by conducting multiple baseline diffusion rate measurements and calculating the standard deviation for the dataset. Because LOD is inversely proportional to the sensitivity, any measures discussed above that can increase the sensitivity of the sensor can improve its LOD. In addition to modifying the parameters shown in Equation (1), the lengths of time for each stage of the measurement cycle could also affect the LOD. The length of the purge stage is important because it needs to be long enough to fully remove the CO_2_ buildup from the system before the measurement stage begins. Increasing the duration of the recirculation stage can increase the repeatability (decrease the noise) of the measurement, so it can improve the LOD. However, increasing the recirculation duration will make the measurement cycles longer. In the tests described in this paper, the flush stage lasted 30 s and the recirculation stage lasted 60 s. So each measurement cycle lasted 1.5 min. For most applications, 1.5‐min measurement intervals are fast enough, but for applications where accuracy is the highest priority, the duration of the recirculation can be increased for better accuracy. At the above conditions, the LOD was calculated to be 0.04% for Bravo‐01, 0.13% for Bravo‐04%, and 0.05% for K30. As mammalian cell cultures are usually maintained at 5% CO_2_, the dCO_2_ in bacteria and yeast fermentations can be higher due to faster growth rate, the described dCO_2_ sensor is sensitive enough for monitoring dCO_2_ concentration in fermentations.

### 3.2 Shake flask monitoring

**Figure 5:**
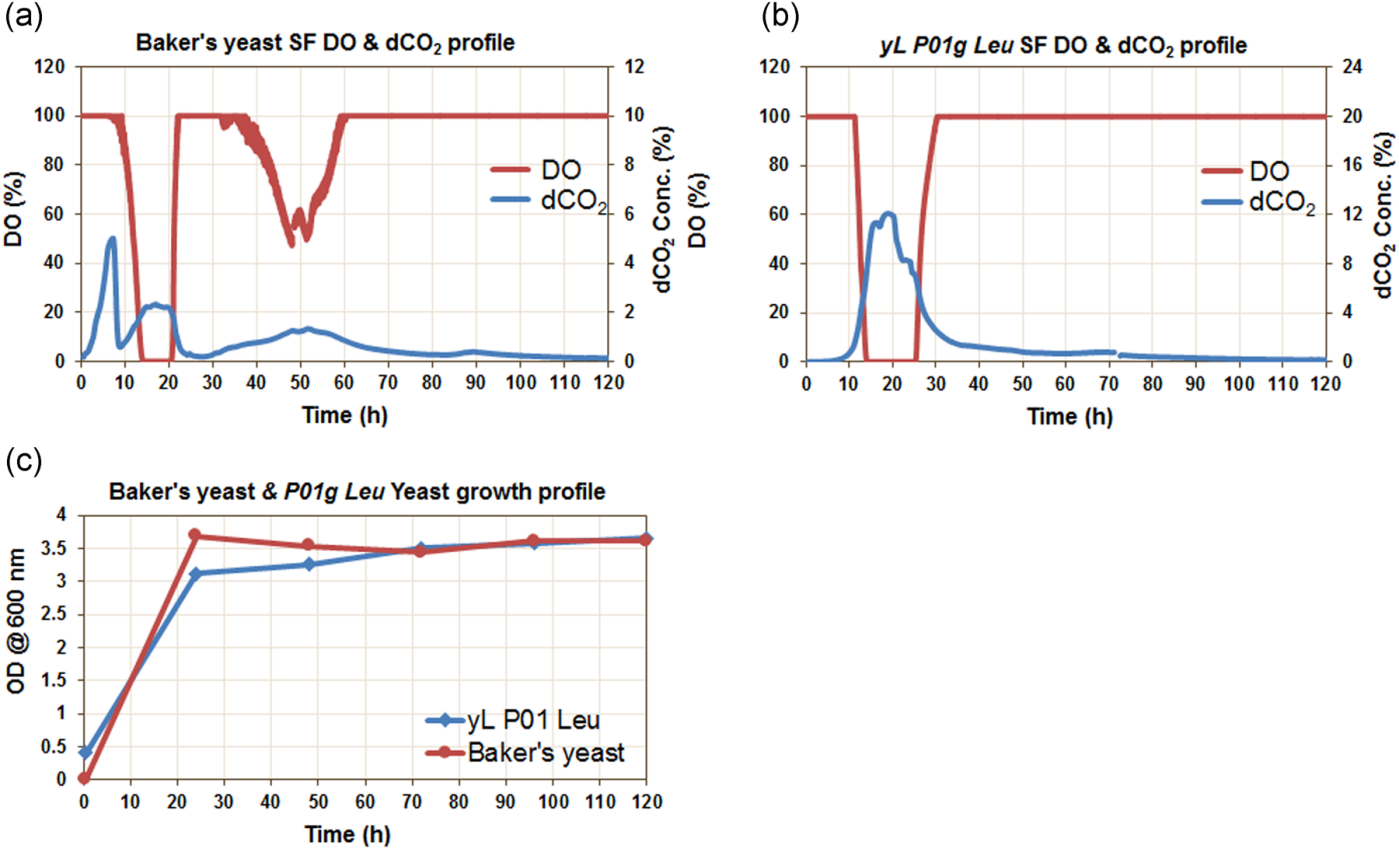
Fermentation profiles monitored in shake flask cultivation. (a) Dissolved oxygen (DO) and dissolved carbon dioxide (dCO_2_) profile for batch baker's yeast culture; (b) DO and dCO_2_ profile for batch *Yarrowia lipolytica P01g‐Leu* yeast culture; (c) optical density (OD) profile for both yeast strains [Color figure can be viewed at wileyonlinelibrary.com]

Experiments were performed using baker's yeast and *Y. lipolytica P01g‐Leu* strain at shake flask scale in batch mode using YPD media. Process data for these preliminary experiments were successfully collected for multiple parameters, including DO, dCO_2_, and optical density (OD). The experiments were started with ∼0.2 and 0.5 initial optical density for baker's yeast and yeast *Y. lipolytica P01g‐Leu*, respectively. Both cultures were grown at 30°C at 250 rpm. The profiles are depicted in Figure 5. It was observed that there was a sharp rise in dCO_2_ concentration in the baker's yeast culture without a concomitant drop in O_2_ consumption. In contrast, the *Y. lipolytica P01g‐Leu* strain showed a steady increase in dCO_2_ concentration with a decline in DO concentration (Figure 5a–c). Figure 5c shows the overlap of the dCO_2_ trend for both yeasts where the significant distinction in their metabolic pattern can be seen. The initial sharp rise in dCO_2_ concentration (first 7 hr) in the case of baker's yeast was probably due to the Crabtree effect where respire‐fermentative glucose metabolism led to the production of CO_2_ and ethanol (Chopda, Rathore, & Gomes, 2013; Chopda, Rathore, & Gomes, 2015; Persad, Chopda, Rathore, & Gomes, 2013). Thereafter, dCO_2_ concentration decreased indicating exhaustion of glucose. From 9 to 20 hr, there was around 2% increase in dCO_2_ concentration which indicates that the produced ethanol was being consumed (Figure 5a). In contrast, the yeast *Y. lipolytica P01g‐Leu* followed a respiratory metabolism and therefore, dCO_2_ rose steadily with a concomitant drop in DO concentration. The overall dCO_2_ concentration was higher in yeast *Y. lipolytica P01g‐Leu* compared with baker's yeast. As shown in Figure 5c, the fermentation period after 48 hr showed a higher OD in baker's yeast, which was around 3.5 even after starting with a lesser initial OD (the first data point was somewhat fluctuating due to the small number and probably sampling error), compared with the OD in *Y. lipolytica P01g‐Leu* yeast, which was around 3.3 even after starting with a higher initial OD of 0.5. This may suggest a higher CO_2_ concentration (~12%) in the case of *Y. lipolytica P01 Leu* yeast might have inhibited cell growth compared with the baker's yeast fermentation where dCO_2_ concentration reached only 5%. This is in accordance with various literature data that have shown a similar inhibitory impact of higher CO_2_ levels (Blombach & Takors, 2015). In addition, a similar distinct dCO_2_ pattern in the Crabtree positive and negative strains was observed when measured in headspace by the RAMOS system (Anderlei et al., 2004).

### 3.3 dCO_2_ control by controlled glucose feeding

The concentration of glucose is critical in the baker's yeast culture as excessive glucose may turn on the Crabtree effect even in oxidative conditions. As glucose metabolism yields to biomass, CO_2_ and water, we gave the target setpoint for the dCO_2_ by keeping glucose in feedback control (on–off control). In the first condition, we controlled the dCO_2_ levels at 10% for 4–8 hr using 400 g/L glucose and then turned off the controller for the remaining process duration. In other conditions, we controlled the dCO_2_ levels at 10% and 6% for 4–24 hr using 400 and 200 g/L glucose feed concentration, respectively. The choice of maintaining a set of dCO_2_ values using a certain concentration of glucose solution has been taken from our preliminary experimentation (data not included) while testing the sensor.

**Figure 6:**
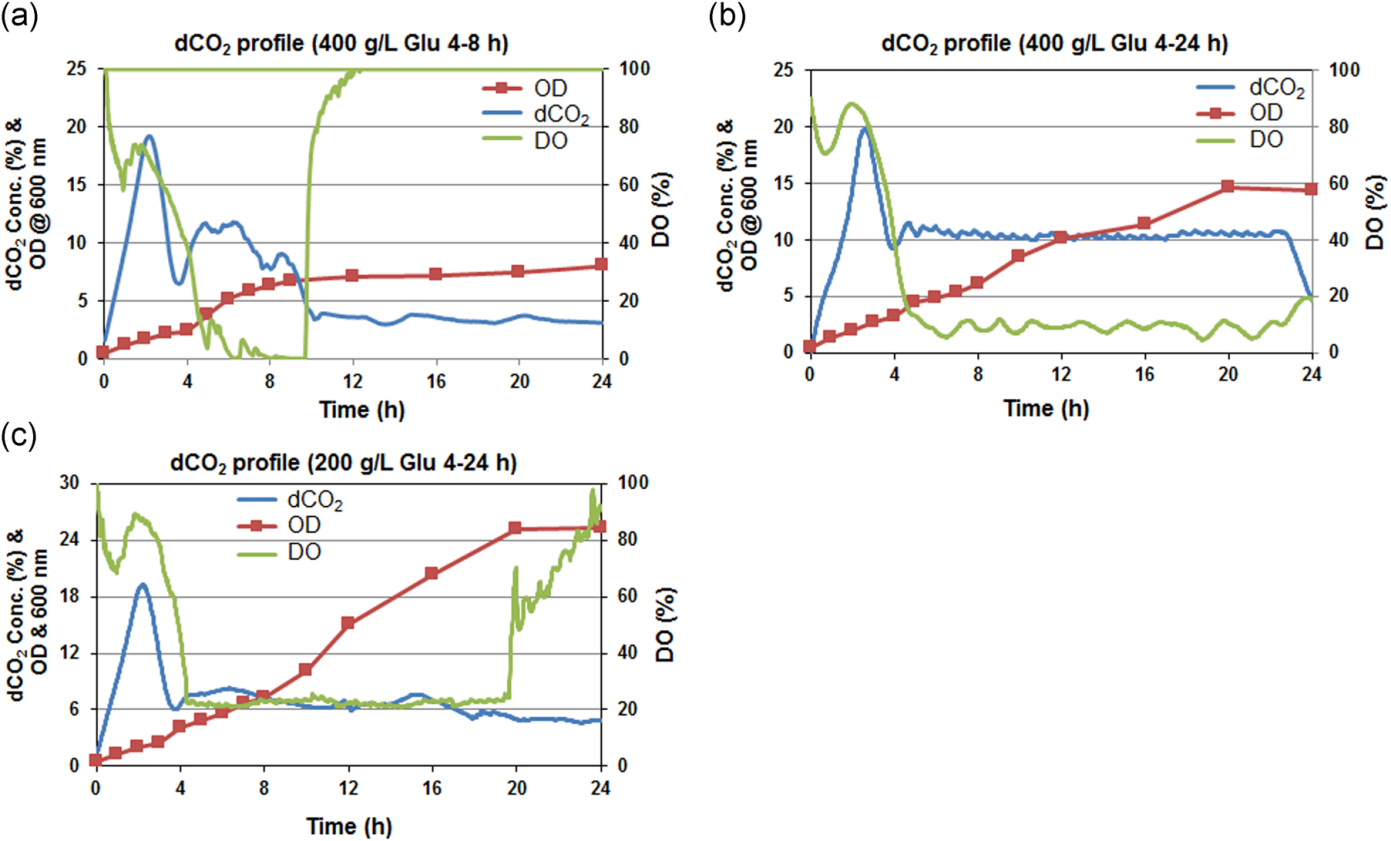
Fermentation process profiles monitored in recombinant baker's yeast culture in a mini bioreactor. (a) Dissolved oxygen (DO), optical density (OD), and dissolved carbon dioxide (dCO_2_) profile for dCO_2_ at 10% 4–8 hr with 400 g/L glucose feed; (b) DO, OD, and dCO_2_ profile for dCO_2_ at 10% 4–24 hr with 400 g/L glucose feed; (c) DO, OD, and dCO_2_ profile for dCO_2_ at 6% 4–24 hr with 200 g/L glucose feed [Color figure can be viewed at wileyonlinelibrary.com]

From Figure 6a–c we can see that the feedback control was able to maintain the dCO_2_ levels around the set target. The experiment in which feeding was performed for only 4–8 hr showed that dCO_2_ levels oscillated at around 10% and thereafter declined as the feeding was stopped (Figure 6a). This condition resulted in lower biomass concentration and reached only OD around 8 (Figure 6a). The experiment in which dCO_2_ maintained at around 10% (4–24 hr), DO remain relatively at lower levels (5–10%) as shown in Figure 6b and OD reached to 14.5. In the third case, we reduced the concentration of the feed solution and set the dCO_2_ levels to around 6%. With controlled glucose addition, dCO_2_ remained well controlled at the desired set point of 6%, which led to significantly higher biomass (OD reached 25.2) as shown in Figure 6c). The biomass yield over glucose in the case of 400 g/L glucose fed (for 4–24 hr) experiments was estimated to be 0.47 OD/g‐glucose while in the case of 200 g/L glucose fed (for 4–24 hr), it was estimated to be 1.0 OD/g‐glucose. With a 4% lower target dCO_2_ concentration, the biomass yield was increased by 114% (Figure 6a–c). This can be explained by the fact that with the condition in which 10% dCO_2_ was maintained, the more glucose pumped into the bioreactor in an oxidative condition might have led to more ethanol formation (Crabtree effect) as a byproduct, which resulted in low biomass concentration (OD = 14.5). In contrast, the other condition in which dCO_2_ was maintained to only 6% was able to maintain the culture in the respiratory regime and resulted in more biomass. Thus, a dCO_2_‐based feeding strategy can allow us to drive the bioprocess in a certain metabolic path. It is very important to track dCO_2_ concentration through various stages of biomanufacturing from inoculum development in shake flask to seed generation at the benchtop bioreactor scale to industrial production scale to monitor culture conditions.

## 4 DISCUSSION

DO‐based control strategies are more popular in bioprocessing because oxygen is considered as an important substrate in metabolism. In addition, robust sensors to monitor DO in real‐time are available at various scales of operation (Chopda, Pathak, Batra, Gomes, & Rathore, 2016; Chopda et al., 2013; Priyanka, Roy, Chopda, Gomes, & Rathore, 2019). However, due to the mass transfer limitation in bioprocesses, pure oxygen must be supplied to fulfill the requirement of DO (Carloni et al., 2009; Priyanka et al., 2019). This often leads to sensor responses at the extreme of their detection limits (either 0 or 100) resulting in oscillation or drift in the signal. For example, in Figure 5, it is hard to predict the existence of the Crabtree metabolism of the organism solely from DO response. In the growth phase, the DO signal went to 0 value and when the cells entered the stationary phase, the DO signal rose to 100% (Figure 5). On the other hand, dCO_2_ provides a more nuanced response that can be correlated to biomass production. This is due to CO_2_ being the final product of respiration both in microbial as well as in mammalian processes. It has a strong impact on cell metabolism in multiple ways as discussed in Section 1 of this paper. Thus, the real‐time dCO_2_ profile can give the true metabolic signature of a process. However, until now, CO_2_ is mostly monitored in the exhaust gas, which itself cannot give real‐time culture broth conditions as the information captured is filtered through the headspace. This means there exists an inherent lag in relaying the dCO_2_ concentrations to the gaseous phase CO_2_ (Chopda et al., 2015). Very few sensors are available in the market which can measure dCO_2_ concentration, and most will not fit in small‐scale systems such as shake flasks or mini bioreactors.

Here we have presented the application of a novel, noninvasive, rate‐based technique for monitoring dCO_2_ in bioprocess. By Fick's law of diffusion, the diffusion rate should be proportional to the concentration of dCO_2_ in the broth. We have shown that by measuring the initial diffusion rate we were able to determine the partial pressure of dCO_2_ in the culture. The technique could be readily automated, and measurements could be made in minutes. It needs to be mentioned that CO_2_ exists in several different forms when it is dissolved in water (CO_2_ [aq], H_2_CO_3_, HCO_3_^−^, CO_3_^2−^), dependent on the pH. The developed method measures only CO_2_ (aq), the unreacted form of dissolved CO_2_. When pH changes significantly, the changes in pH should be taken into consideration to accurately determine the CO_2_ evolution rate.

The dCO_2_ sensor was tested in demonstration experiments by growing baker's yeast and *Y. lipolytica* cultures. These results show that distinct changes in the metabolism of an organism can be captured using dCO_2_ signatures, suggesting that the rate‐based method is an effective way to monitor dCO_2_ levels in small‐scale systems. Furthermore, we have tested a control strategy based on dCO_2_ concentration by controlled glucose feeding in the feedback loop. Different dCO_2_ profiles gave very distinct results in terms of biomass formation in baker's yeast mini bioreactor culture. The lowered concentration of dCO_2_ resulted in a significant increase in biomass productivity (Figure 6). The experiment performed for sensor characterization (Figure 5) is the run in which no dCO_2_ control was implemented that resulted in a maximum OD of around 3.5 at around 24 hr. On the other hand, all the dCO_2_ controlled experiments (Figure 6) yielded a higher OD. The highest OD of 25.2 was achieved when dCO_2_ was controlled at 6%, which is almost a seven‐time increase in OD compared with uncontrolled conditions. Many researchers have used off‐gas measurements to estimate the biomass in the reactor. The substrate demand is then determined according to the amount of biomass. However, this method is still an “open loop” control strategy and may lead to a lag in control action (Muthuswamy & Srinivasan, 2003; Valentinotti et al., 2003). Other case studies in the benchtop, as well as large scale bioreactors, emphasize the important role of dCO_2_ monitoring and its significant impact on cell growth and protein production (Mitchell‐Logean & Murhammer, 1997; Mostafa & Gu, 2003).

A similar phenomenon occurring in shake flask culture, where the performance of the shake flask in terms of biomass growth and product concentration was significantly affected by dCO_2_ concentrations, was reported (Takahashi & Aoyagi, 2018a, 2018b). In one of the more interesting findings, researchers have shown that opening the shake flask closure for sampling allows diffusion of gases into the atmosphere, such that CO_2_ concentrations decrease temporarily in the broth as well as in the headspace, thereby significantly impacting the community structure of soil microbes (Takahashi & Aoyagi, 2018b). A study conducted by McIntyre and McNeil (1997) concluded that culture is more vulnerable to CO_2_ inhibition in the lag phase. In addition, during the inoculation step, the culture can experience many pressures due to the significant differences in the environment of a shake flask and in the bioreactor conditions. Monitoring dCO_2_ levels across all scales of biomanufacturing will enable control of the culture environment to prevent any severe shock to the growing cells.

We believe that the studies presented in this paper using our in situ dCO_2_ sensor proves the criticality and importance of monitoring and control of dCO_2_. Monitoring dCO_2_ will not only indicate real‐time culture conditions but also give the critical process information for taking effective control decisions should deviations occur. We also showed that the proposed sensor is flexible in configuration and can be easily fit in shake flasks and other small‐scale mini bioreactors. This will enable us to extract more process information even from small‐scale systems thereby accelerating process development many folds.

## 5 CONCLUSION

Shake flask and the small‐scale fermentation processes are generally hindered by the scarcity of in‐line sensors. We have identified that there is a need to develop a portable dCO_2_ sensor for such small‐scale fermentation due to the significant effect of dCO_2_ on the overall metabolism and the subsequent scale‐up activities. In this manuscript, we present a novel, rate‐based technique for monitoring dCO_2_ in cell cultures by measuring the initial diffusion rate across a silicone sampling tube immersed in the culture media. Demonstration experiments conducted with baker's yeast and *Y. lipolytica* yeast cells in both shake flasks and mini bioreactors show that it can monitor dCO_2_ in real‐time. Using the proposed sensor, we successfully implemented a dCO_2_‐based control scheme, which resulted in significant improvement in process performance. Through the implementation of dCO_2_ based control strategy, we have shown that by controlling dCO_2_ at various levels we can direct the metabolic fate of the process in real‐time. In the future, our efforts will be directed towards using the developed dCO_2_ based control scheme to reduce the disparity that exits across the different scales of operation.
